# Supplementary material for: Conformational biosensors delineate endosomal G protein regulation by GPCRs
Source: Nat Commun. 2026 Feb 18;17:2911. doi: 10.1038/s41467-026-69329-9 (PMC13031922; doi:10.1038/s41467-026-69329-9)
Supplement: Supplementary file 1 — Supplementary Information [file 41467_2026_69329_MOESM1_ESM.pdf]

Supplementary Information

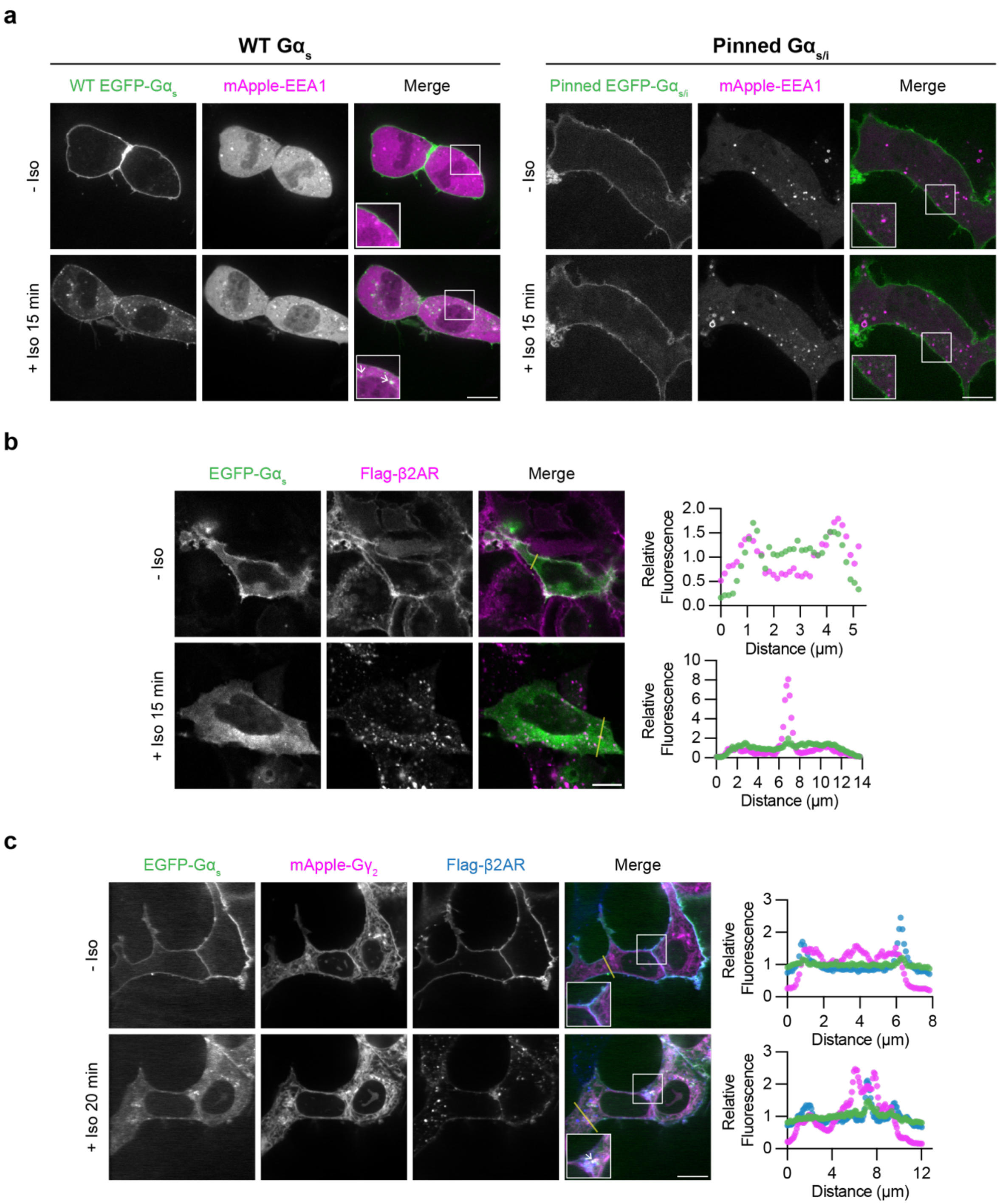

Supplementary Figure 1: Agonist-induced redistribution of Gα$_s$ in live and fixed cells. a) Representative stills from time-lapse confocal microscopy of live HEK293 cells stably expressing Flag-β2AR and transiently expressing either WT EGFP-Gα$_s$ (left) or the membrane-pinned EGFP-Gα$_{s/i}$ chimera (right) and the endosomal marker mApple-EEA1 before or after 15

minutes of Iso (1 μM) treatment. Quantification is shown in Figure 1c. **b)** Left: Representative images of fixed HEK293 cells stably expressing Flag-β2AR and transiently expressing EGFP-$G\alpha_s$ before or after 15 minutes of Iso (1 μM) treatment. Right: Line scans depicting fluorescence intensity along indicated regions. **c)** Left: Additional example of representative confocal images of live HEK293 cells stably expressing Flag-β2AR and transiently expressing EGFP-$G\alpha_s$ and mApple-$G\gamma_2$ before and after 20 minutes of Iso (1 μM) treatment (another example is shown in Figure 1d). Right: Line scans depicting fluorescence intensity along indicated regions. Prior to imaging (a, c) or drug treatment and fixation (b), cells were treated for 10 minutes with an anti-Flag antibody coupled to Alexa Fluor 647 to label surface Flag-β2AR. Cells were co-transfected with either myc-$G\beta_1$ and untagged $G\gamma_2$ or untagged $G\beta_1$ and myc-$G\gamma_2$ (a, b) or untagged $G\beta_1$ (c). Images are representative of at least 3 independent experiments, scale bars are 10 μm, and insets are 1.5x zoom of indicated regions. Arrows indicate examples of colocalization. Source data are provided as a source data file.

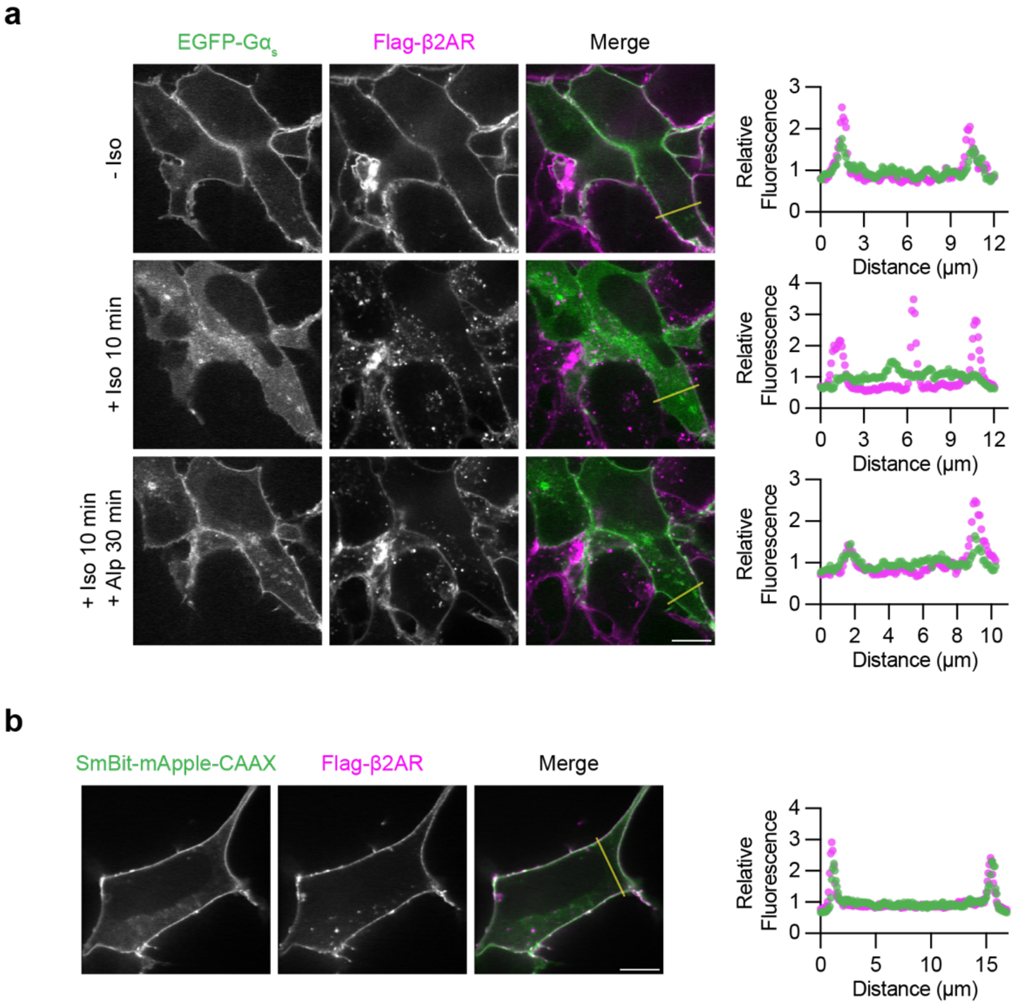

Supplementary Figure 2: Redistribution of Gα_s is reversible. a) Left: Representative stills from time-lapse confocal microscopy of live HEK293 cells stably expressing Flag-β2AR and transfected with EGFP-Gα_s, myc-Gβ_1, and untagged Gγ_2 either before drug treatment, after 10 minutes of Iso (100 nM) treatment, or after 10 minutes of Iso followed by 30 minutes of Alprenolol (Alp, 10 μM) treatment. EGFP-Gα_s channel images are reproduced from Figure 2a. Right: Line scans depicting fluorescence intensity along indicated regions. **b)** Left**:** Representative confocal images of HEK293 cells transfected with SmBit-mApple-CAAX and Flag-β2AR. Right: Line scans depicting fluorescence intensity along indicated regions. Prior to imaging, cells were treated for 10 minutes with an anti-Flag antibody coupled to Alexa Fluor 647 to label surface Flag-β2AR. Images are representative of at least 3 independent experiments. Scale bars = 10 μm. Source data are provided as a source data file.

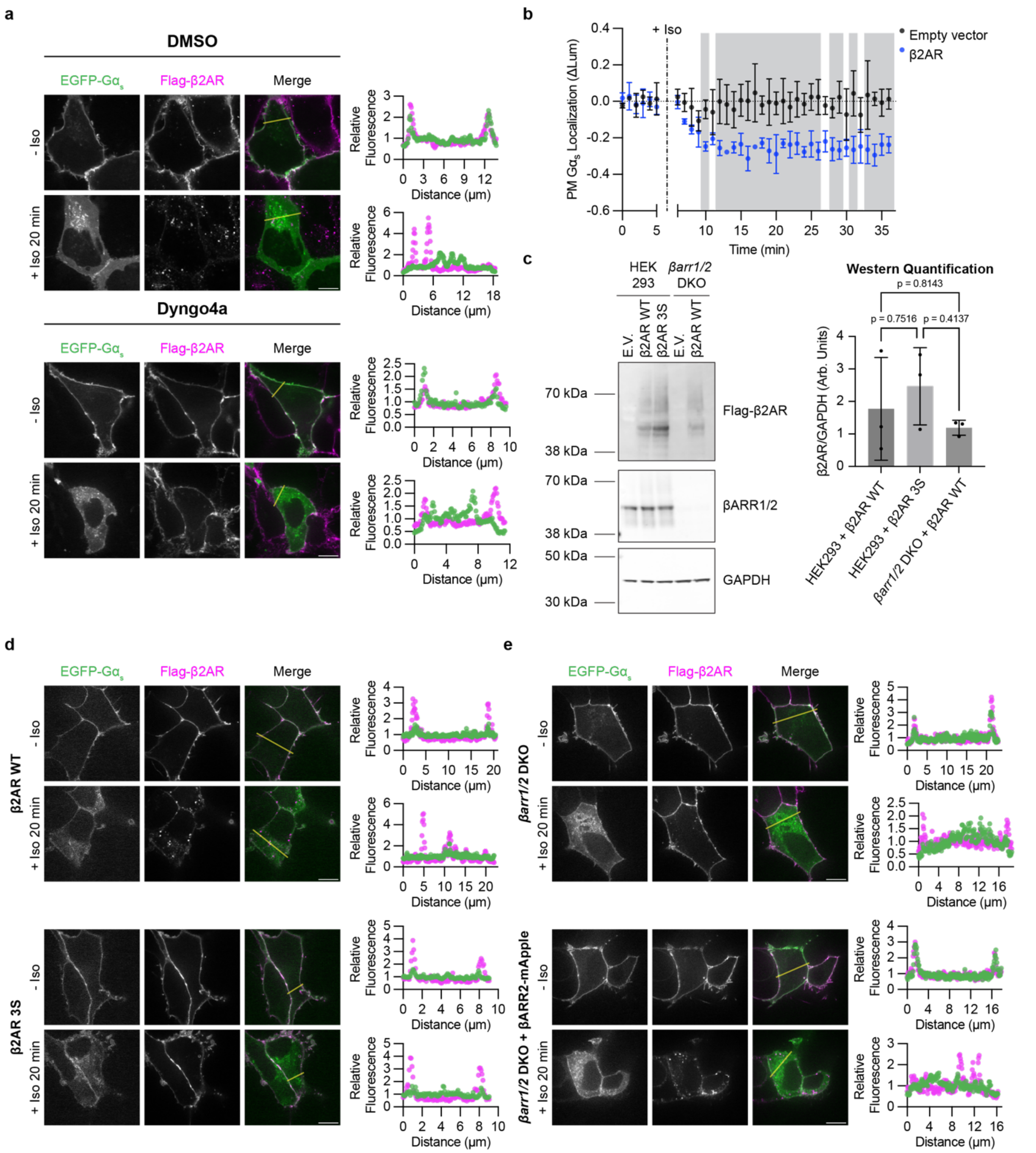

Supplementary Figure 3: Gα$_s$ redistribution is independent of receptor internalization. a) Left: Representative stills from time-lapse confocal microscopy of live HEK293 cells stably expressing Flag-β2AR and transiently expressing EGFP-Gα$_s$ before or after 20 minutes of Iso (1 μM) treatment. Cells were either pretreated with DMSO (top, 0.1 %) or Dyngo4a (bottom, 30 μM) for 25 minutes prior to imaging, and cells were incubated for the final 10 minutes of compound pretreatment with an anti-Flag antibody coupled to Alexa Fluor 647 to label surface Flag-β2AR. Iso was added after 5 minutes of imaging. Scale bars = 10 μm. Right: Line scans depicting fluorescence intensity along indicated regions. **b)** NanoBit bystander assay showing plasma membrane localization of Gα$_s$ in HEK293 cells expressing WT Flag-β2AR (blue) or empty vector (black). Iso (1 μM) was added at 5 minutes. Shaded areas represent time points at

which the difference between the β2AR and empty vector conditions is significant ($p < 0.05$, determined by repeated measures ANOVA with Sidak's multiple comparisons test, see source data). **c)** Left: Representative western blots of HEK293 cells expressing Flag-β2AR WT or Flag-β2AR 3S, or *βarr1/2* DKO cells expressing WT β2AR. Right: Densitometry analysis of western blots. Significance was determined by ordinary one-way ANOVA followed by Tukey's multiple comparisons test. **d)** Left: Representative confocal images of HEK293 cells transiently coexpressing either WT Flag-β2AR (top) or Flag-β2AR-3S (bottom) and EGFP-$G\alpha_s$ before or after 20 minutes of Iso (100 nM) treatment. Cells were co-transfected with untagged $G\beta_1$ and mApple-$G\gamma_2$. Right: Line scans depicting fluorescence intensity along indicated regions. **e)** Left: Representative confocal images of *βarr1/2* DKO HEK293 cells transiently coexpressing WT Flag-β2AR, EGFP-$G\alpha_s$, and either mApple control (top) or βARR2-mApple (bottom) before or after 20 minutes of Iso (100 nM) treatment. Cells were co-transfected with untagged $G\beta_1$ and myc-$G\gamma_2$. Right: Line scans depicting fluorescence intensity along indicated regions. In panels d and e, cells were treated prior to imaging for 10 minutes with an anti-Flag antibody coupled to Alexa Fluor 647 to label surface Flag-β2AR. Images are representative of at least 3 independent experiments, and scale bars are 10 μm. Data are represented as mean ± S.D. of 3 independent experiments. Source data are provided as a source data file.

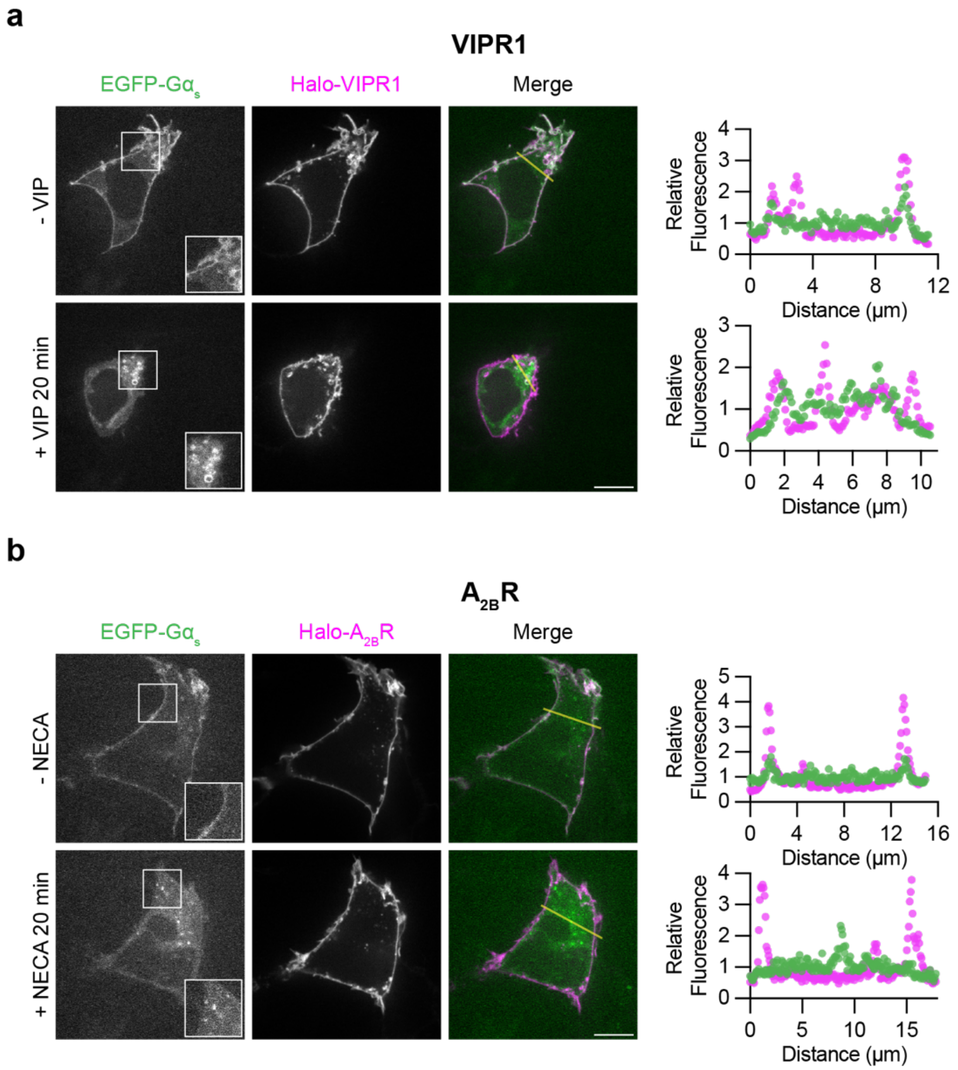

Supplementary Figure 4: VIPR1 and $A_{2B}R$ trigger redistribution of $G\alpha_s$ to internal compartments. a,b) Left: Representative stills from time-lapse confocal microscopy of live HEK293 cells expressing EGFP-$G\alpha_s$ and either Halo-VIPR1 **(a)** or Halo-$A_{2B}R$ **(b)** before and after 20 minutes of VIP (1 µM, a) or NECA (100 µM, b) treatment. Insets (1.5x) show examples of internal punctate localization of EGFP-$G\alpha_s$ after receptor activation. Right: Line scans depicting fluorescence intensity along indicated regions. Prior to imaging, cells were treated for 10 minutes with 200 nM JF_{635}i-HTL to label surface receptors, and cells in panel b were co-treated with 10 µM H89 to prevent agonist-induced contractility. Cells were co-transfected with $G\beta_1$ and mApple-$G\gamma_2$. Images are representative of 4 (panel a) or 3 (panel b) independent experiments, and scale bars = 10 µm. Source data are provided as a source data file.

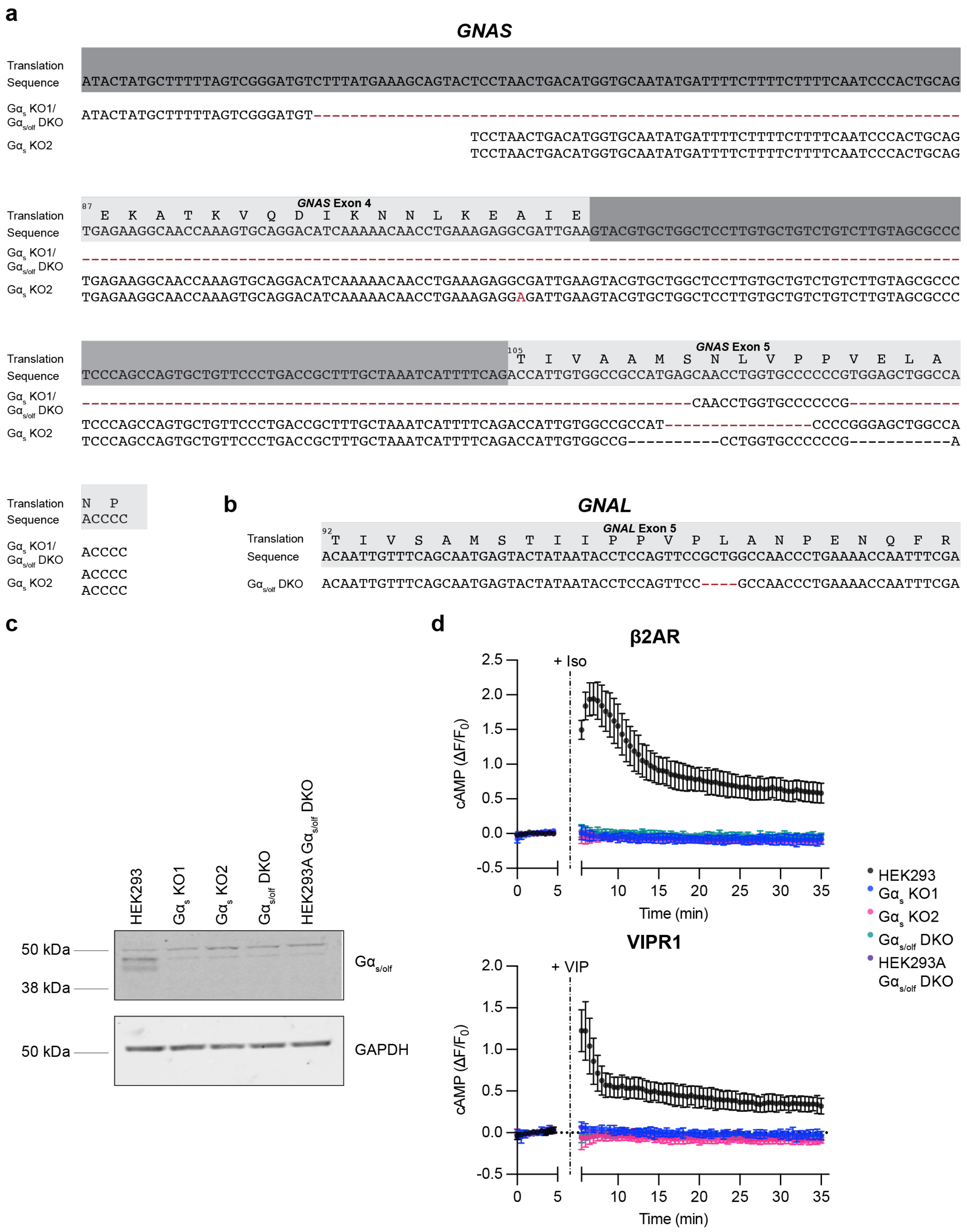

Supplementary Figure 5: Characterization of Gα$_s$ KO and Gα$_{s/olf}$ DKO cells used in this study. a) Sequence alignments of *GNAS* locus (encoding Gα$_s$) demonstrating genetic modifications of novel monoclonal Gα$_s$ KO and Gα$_{s/olf}$ DKO cell lines generated in this study. **b)** Sequence alignments of *GNAL* locus (encoding Gα$_{olf}$) demonstrating genetic modifications of

the novel monoclonal G$\alpha_{s/olf}$ cell line generated in this study. **c)** G$\alpha_{s/olf}$ western blot of Gα_s KO and G$\alpha_{s/olf}$ DKO cells confirming knockout of G$\alpha_{s/olf}$ protein. HEK293A G$\alpha_{s/olf}$ cells were included as a positive control for non-specific antibody binding. Blot is representative of 3 independent experiments. **d)** Green cADDis cAMP assays of HEK293 parental cells and monoclonal Gα_s and G$\alpha_{s/olf}$ cells after endogenous β2AR (top) or VIPR1 (bottom) activation. Cells were treated with Iso (100 nM) or VIP (1 μM) at 5 minutes. HEK293A G$\alpha_{s/olf}$ cells were included as a positive control for loss of cAMP response. Data are presented as mean ± S.D. of either 5 (HEK293) or 3 (all other cell lines) independent experiments. Source data are provided as a source data file.

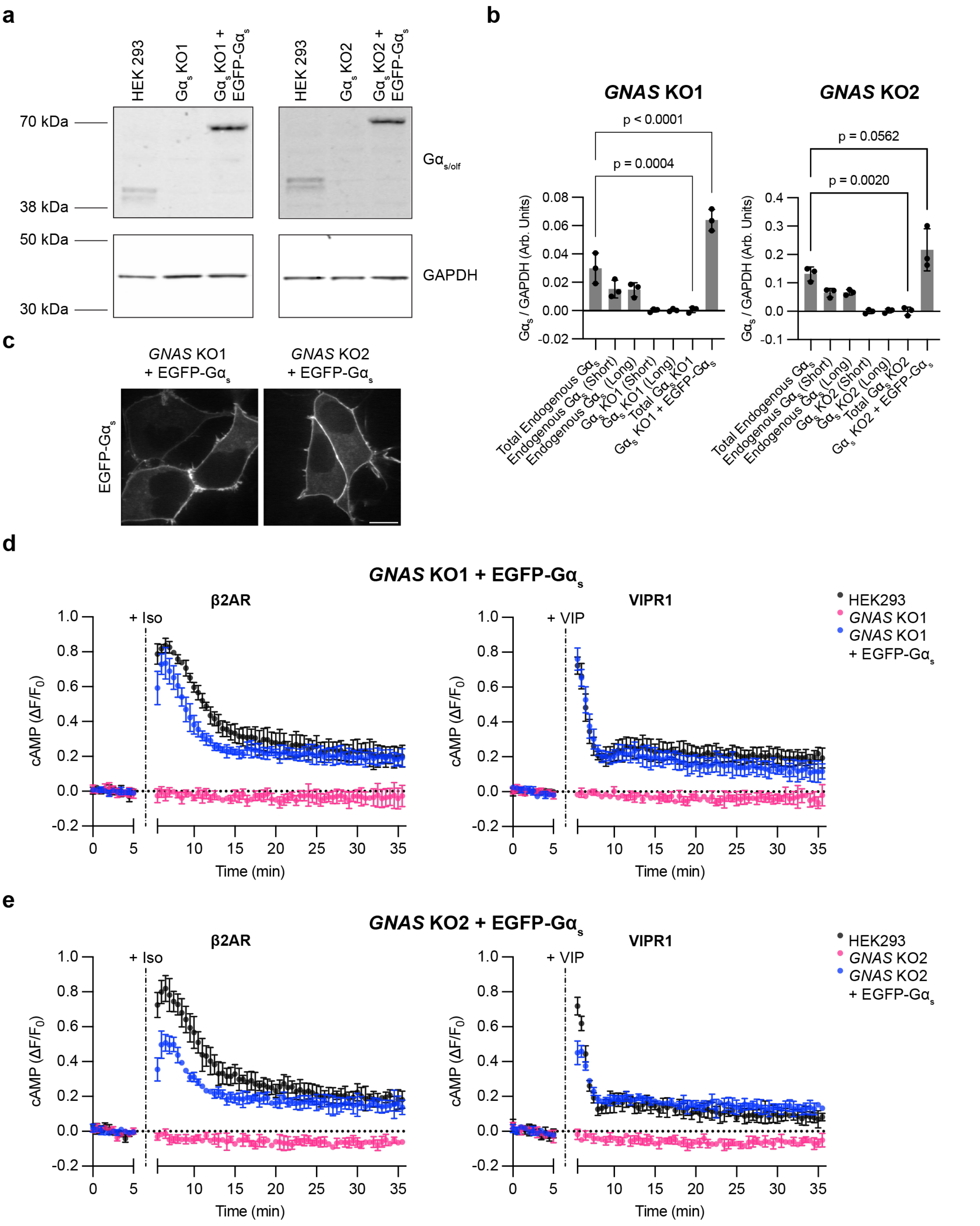

Supplementary Figure 6: Characterization of Gα_s KO + EGFP-Gα_s rescue cells used in this study. a) Representative western blots of parental HEK293, Gα_s KO1 cells, and Gα_s KO1 + EGFP-Gα_s rescue cells (left) or HEK293 cells, Gα_s KO2 cells, and Gα_s KO2 + EGFP-Gα_s rescue cells (right). **b)** Densitometry analysis of western blots from a). Data are shown as mean ± S.D.

of 3 independent experiments. Significance was determined by one-way ANOVA followed by Tukey's multiple comparisons test. **c)** Confocal micrographs of live Gα_s KO1 or KO2 + EGFP-Gα_s rescue cells demonstrating plasma membrane localization of EGFP-Gα_s. Images are representative of 2 independent experiments. **d)** Red cADDis cAMP assays of HEK293 parental cells, Gα_s KO1, and Gα_s KO1 + EGFP-Gα_s rescue cells after activation of endogenous β2AR (left) or VIPR1 (right). **e)** Red cADDis cAMP assays of HEK293 parental cells, Gα_s KO2, and Gα_s KO2 + EGFP-Gα_s rescue cells after activation of endogenous β2AR (left) or VIPR1 (right). For data in d) and e), Iso (100 nM) or VIP (1 μM) were added at 5 minutes, and data are shown as mean ± S.D. of 3 (*GNAS* KO2 and *GNAS* KO2 + EGFP-Gα_s) or 4 (all other cell lines) independent experiments. Source data are provided as a source data file.

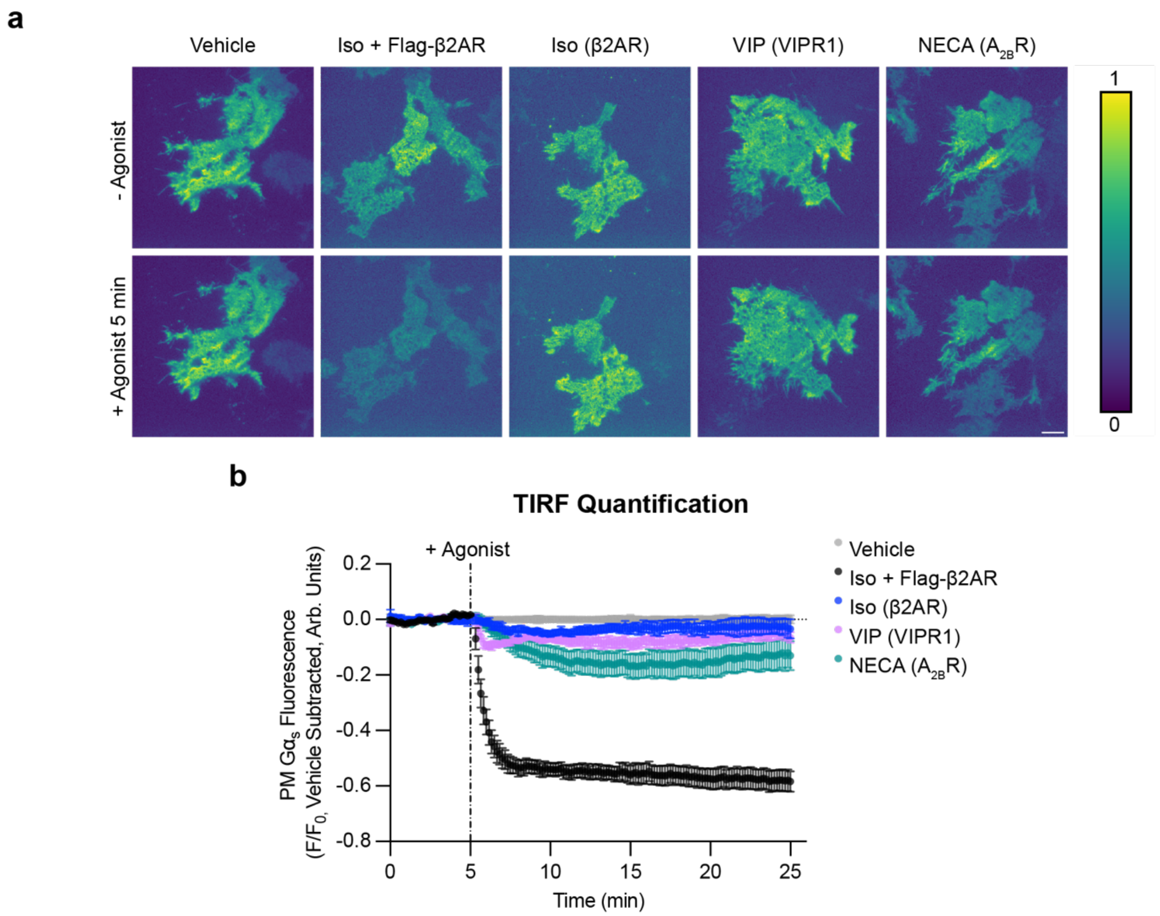

Supplementary Figure 7: Endogenous GPCR activation triggers Gα$_s$ redistribution in an independent clone of EGFP-Gα$_s$ KO rescue cells. a) Representative stills from time-lapse TIRF microscopy of Gα$_s$ KO2 + EGFP-Gα$_s$ rescue cells stably expressing EGFP-Gα$_s$ before or after 5 minutes of agonist treatment to activate endogenously expressed GPCRs (β2AR, VIPR1, A$_{2B}$R). Images are represented as heat maps normalized to t = 3 minutes before drug addition for each individual movie. Cells were treated with either vehicle, Iso (1 µM, ± overexpression of Flag-β2AR), VIP (1 µM), or NECA (20 µM). Scale bar = 10 µm. **b)** Quantification of Gα$_s$ fluorescence from TIRF movies depicted in a). The average of vehicle control movies (n = 4) at each time point was subtracted before plotting data. Data are represented as mean ± S.E.M. of individual movies (n = 4 movies from 4 experiments (2 to 6 cells per movie)). Significance was determined by repeated measures 2-way ANOVA with Dunnett's multiple comparisons test (see source data for p values). Source data are provided as a source data file.

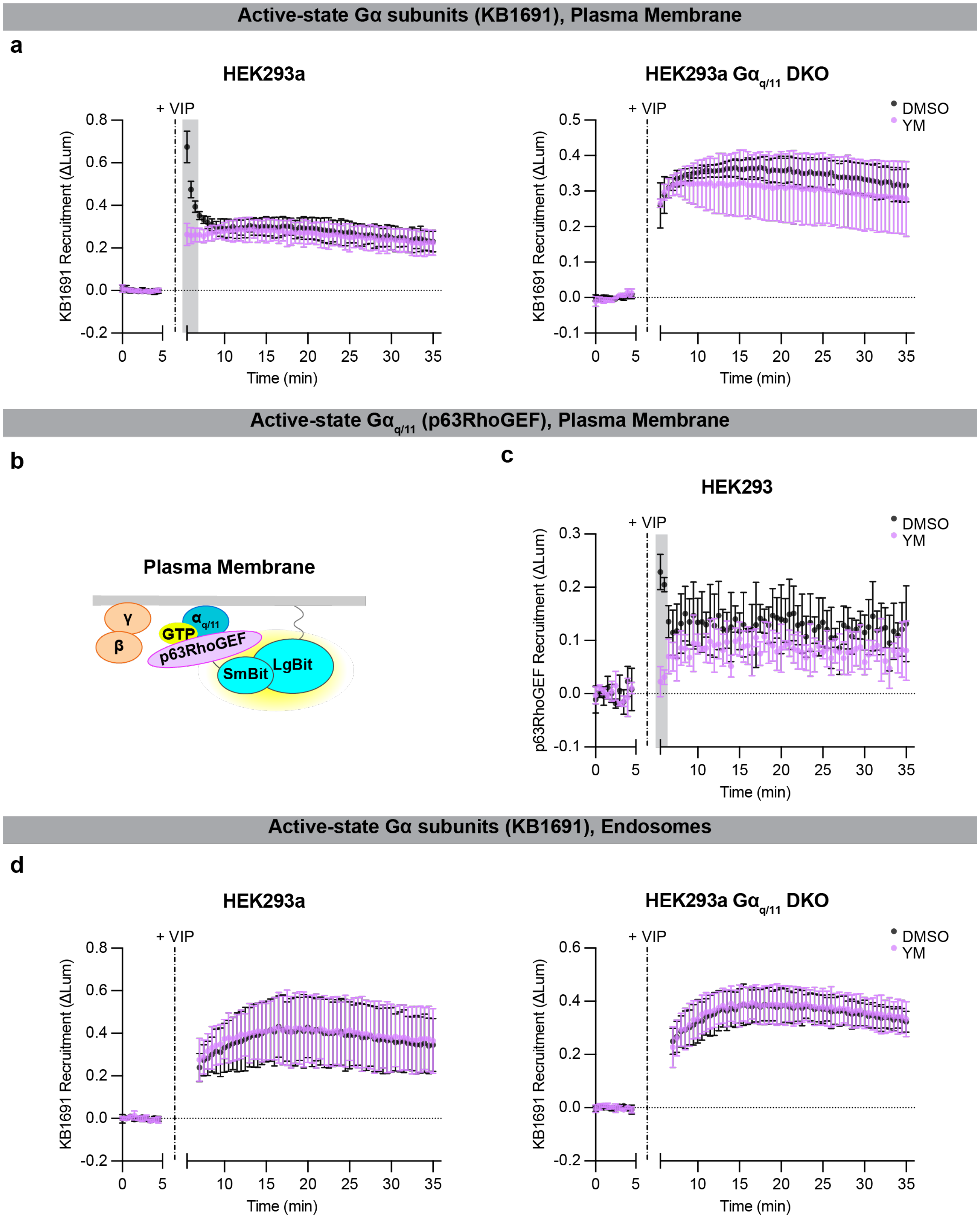

Supplementary Figure 8: Resolving the non-G$_s$ component of active-state Gα production as Gα$_{q/11}$. a) NanoBit bystander assays showing recruitment of KB1691 to the plasma membrane in both HEK293a parental cells (left) and HEK293a Gα$_{q/11}$ DKO cells (right) expressing Halo-VIPR1 and pretreated with either DMSO (0.1 %) or YM-254890 (1 μM, 30 minutes). VIP (1 μM) was added after 5 minutes. **b)** Schematic of p63RhoGEF (active-state Gα$_{q/11}$ biosensor) endosome NanoBit bystander assay. **c)** NanoBit bystander assay showing

recruitment of p63RhoGEF to the plasma membrane in HEK293 cells expressing Halo-VIPR1 and pretreated with either DMSO (0.1 %) or YM-254890 (1 μM, 30 minutes). **d)** NanoBit bystander assays showing recruitment of KB1691 to endosomes in both HEK293a parental cells (left) and HEK293a $G\alpha_{q/11}$ DKO cells (right) expressing Halo-VIPR1 and pretreated with either DMSO (0.1 %) or YM-254890 (1 μM, 30 minutes). VIP (1 μM) was added after 5 minutes. For all panels, shaded areas represent time points at which the difference between DMSO- and YM-treated cells are statistically significant ($p < 0.05$, determined by repeated measures 2-way ANOVA with Sidak's multiple comparisons test, see source data). Data are shown as mean ± S.D. of 3 (panel a, left, panel c, and panel d, left) or 4 (panels a and b, right) independent experiments. Source data are provided as a source data file.

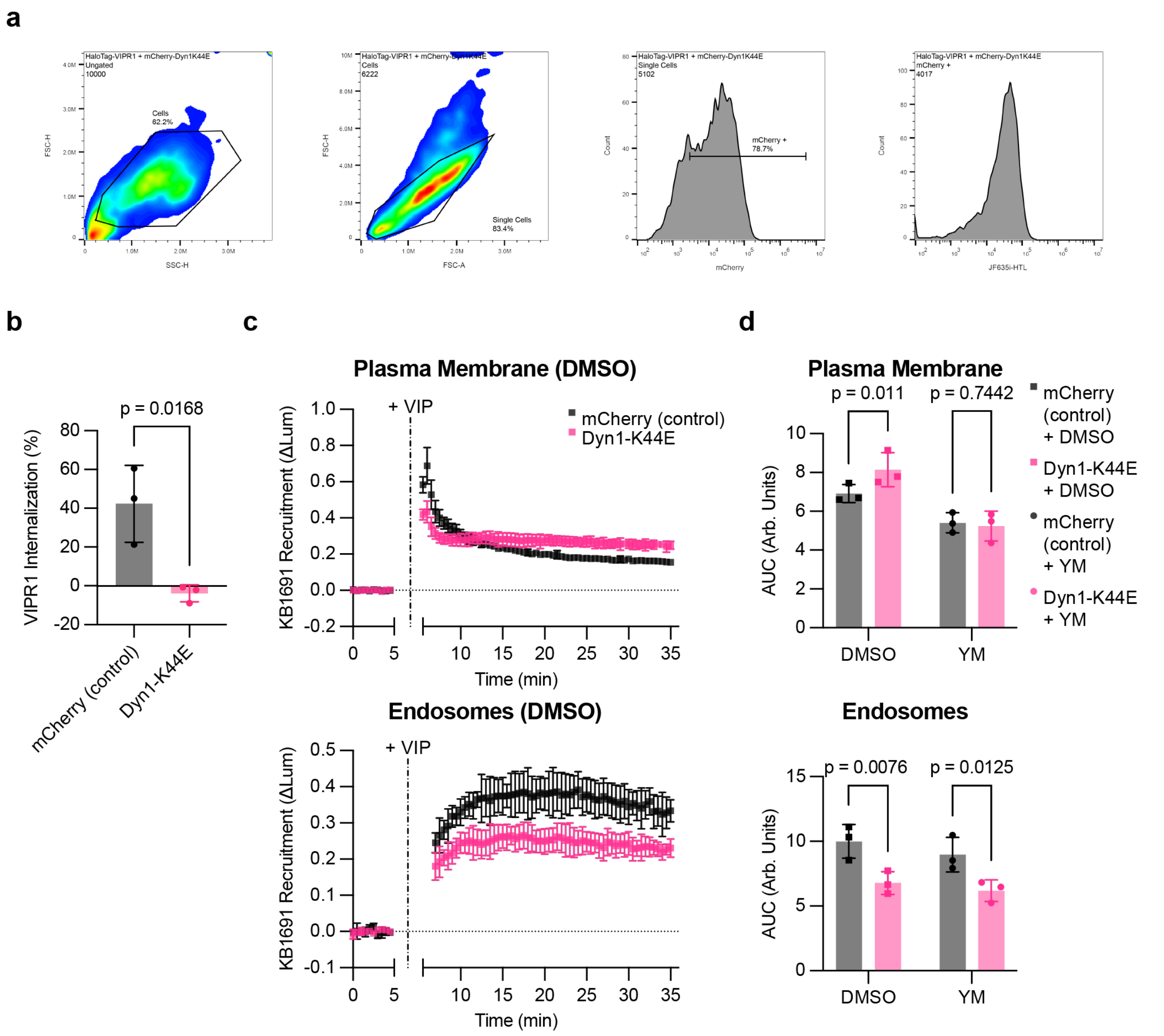

Supplementary Figure 9: Production of active-state Gα$_s$ on endosomes by VIPR1 is endocytosis-dependent. a) Representative gating strategy for VIPR1 flow cytometry internalization experiments in panel b. **b)** Internalization of Halo-VIPR1 (as measured by flow cytometry) after 30 minutes of VIP (1 μM) treatment in VIPR1 knockout cells stably expressing Halo-VIPR1 and transiently expressing either mCherry or mCherry-Dyn1-K44E. Significance determined by unpaired, two-tailed t test. **c)** DMSO control curves for KB1691 plasma membrane (top) or endosomes (bottom) NanoBit bystander assay data shown in Figure 5ef. HEK293 cells expressing Halo-VIPR1 were pretreated with DMSO (0.1 %, 30 minutes) and VIP (1 μM) was added after 5 minutes. Cells were prepared in parallel with cells pretreated with YM-254890 (Fig. 5ef) and data were collected in the same plate. **d)** Area under the curve of NanoBit data in panel a (DMSO) and Figure 5ef (YM). YM bars are reproduced from Figure 5ef. Significance was determined by repeated measures 2-way ANOVA with Sidak's multiple comparisons test (see source data). Data are shown as mean ± S.D. of 3 independent experiments. Source data are provided as a source data file.

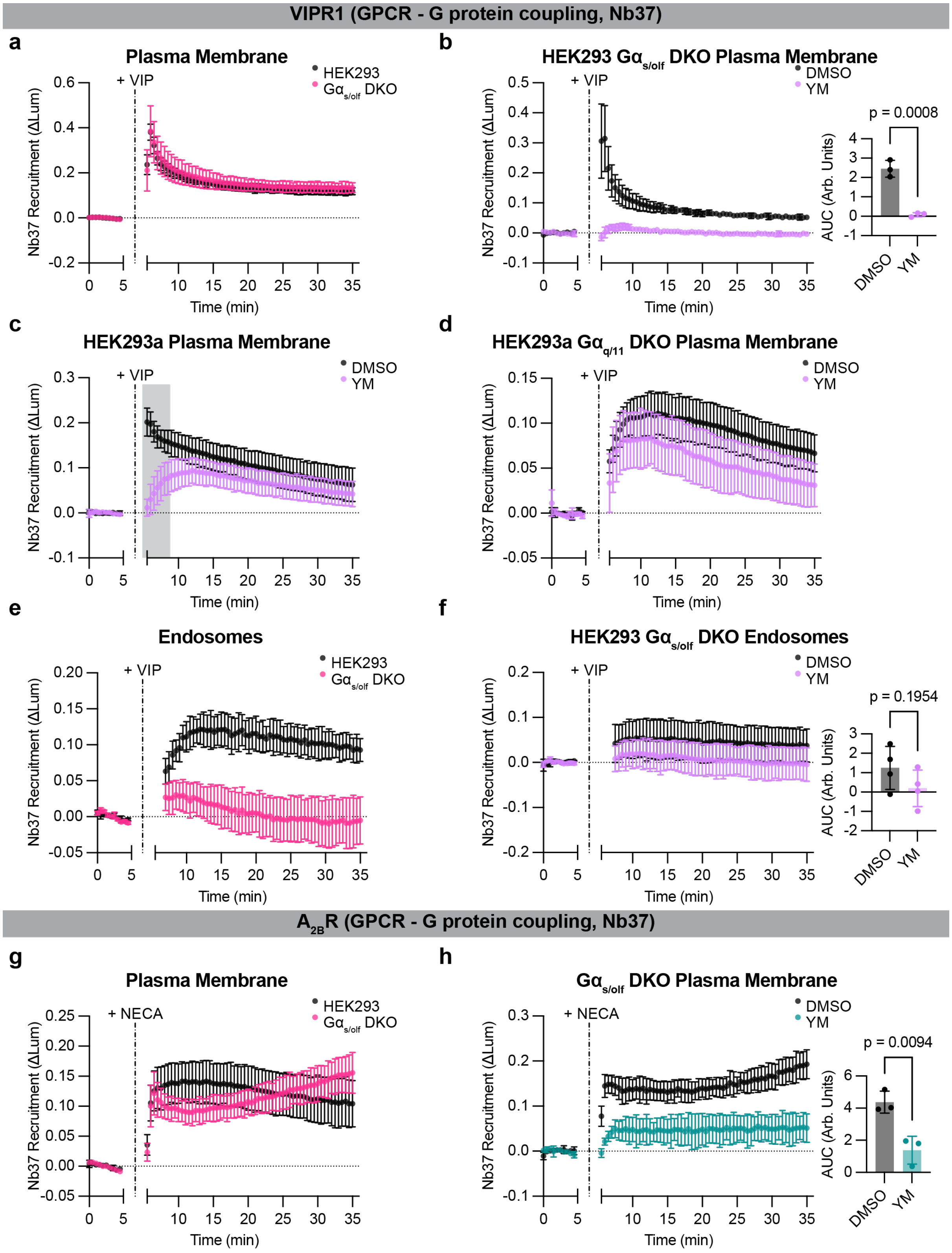

Supplementary Figure 10: Resolving G_s and $G_{q/11}$ components of GPCR coupling detected by Nb37. a) NanoBit bystander assay showing recruitment of Nb37 to the plasma membrane in both HEK293 parental cells and $G\alpha_{s/olf}$ DKO cells expressing Halo-VIPR1. VIP (1

μM) was added after 5 minutes. **b)** Left: NanoBit bystander assay showing recruitment of Nb37 to the plasma membrane in $G\alpha_{s/olf}$ DKO cells expressing Halo-VIPR1 and pretreated with either DMSO (0.1 %) or YM-254890 (1 μM, 30 minutes). Right: AUC of time course. VIP (1 μM) was added after 5 minutes. Significance determined by unpaired, two-tailed t test. **c,d)** NanoBit bystander assays showing recruitment of Nb37 to the plasma membrane in either HEK293a parental cells **(c)** or HEK293a $G\alpha_{q/11}$ DKO cells **(d)** expressing Halo-VIPR1. VIP (1 μM) was added after 5 minutes. Shaded areas represent time points at which the difference between DMSO- and YM- treated cells are statistically significant ($p < 0.05$, determined by repeated measures 2-way ANOVA with Sidak's multiple comparisons test, see source data). **e)** NanoBit bystander assay showing recruitment of Nb37 to endosomes in both HEK293 parental cells and $G\alpha_{s/olf}$ DKO cells expressing Halo-VIPR1. VIP (1 μM) was added after 5 minutes. **f)** Left: NanoBit bystander assay showing recruitment of Nb37 to endosomes in $G\alpha_{s/olf}$ DKO cells expressing Halo-VIPR1 and pretreated with either DMSO (0.1 %) or YM-254890 (1 μM, 30 minutes). Right: AUC of time course. VIP (1 μM) was added after 5 minutes. Significance determined by two-tailed unpaired t test. **g)** NanoBit bystander assay showing recruitment of Nb37 to the plasma membrane in both HEK293 parental cells and $G\alpha_{s/olf}$ DKO cells expressing Halo-A_{2B}R. NECA (100 μM) was added after 5 minutes. **h)** Left: NanoBit bystander assay showing recruitment of Nb37 to the plasma membrane in $G\alpha_{s/olf}$ DKO cells expressing Halo-A_{2B}R and pretreated with either DMSO (0.1 %) or YM-254890 (1 μM, 30 minutes). Right: AUC of time course. NECA (100 μM) was added after 5 minutes. Significance was determined by unpaired, two-tailed t test. Data are shown as mean ± S.D. of 3 (panels a-c, g-h) or 4 (panels d-f) independent experiments. Source data are provided as a source data file.

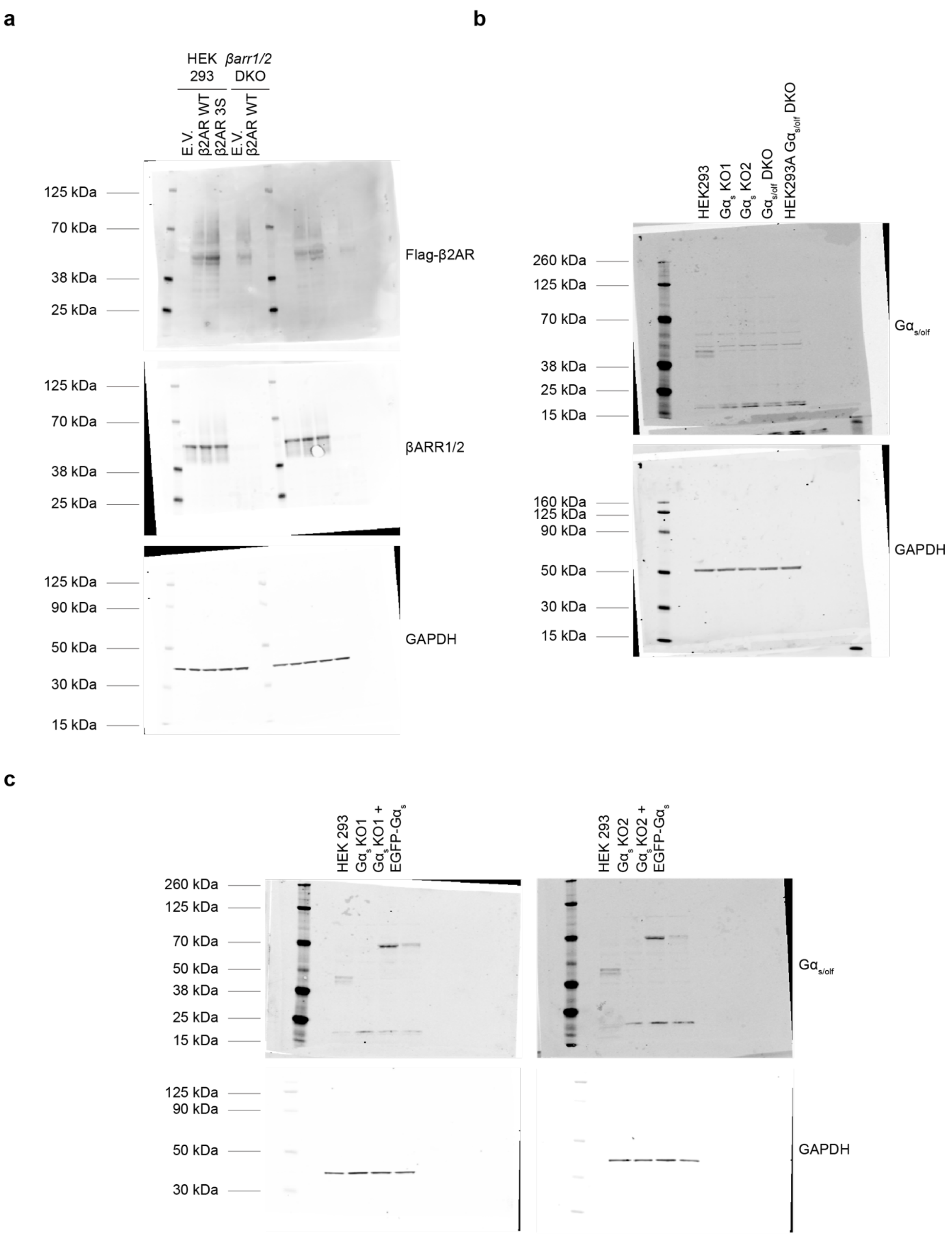

Supplementary Figure 11: Uncropped western blots. a) Uncropped western blots from Supplementary Figure 3c. **b)** Uncropped western blots from Supplementary Figure 5c. **c)** Uncropped western blots from Supplementary Figure 6a.

Supplementary Table 1: DNA constructs used in this study

Construct	Source
pcDNA3-EGFP	This study: insertion of EGFP sequence from EGFP-N1 (Takara Bio) into pcDNA3.
pcDNA3-EGFP-GNAS	This study: replacement of residues 72-81 of HA-GNAS in pcDNA3-HA-GNAS (Dai et al. *Cell* 2022, 10.1016/j.cell.2022.09.019) with EGFP by InFusion cloning (Takara Bio).
pcDNA3-SS-Flag-β2AR	From Cao et al. *Nature* 1999, 10.1038/45816.
pcDNA3-SS-Flag-β2AR-3S	From Irannejad et al. *Nature* 2013, 10.1038/nature12000.
pcDNA3.1-Myc-GNB1	From R. Irannejad.
pcDNA3.1-GNG2	From R. Irannejad.
pCAGGS-GNB1-T2A-MYC-GNG2	This study: Insertion of GNB1-T2A, and myc-GNB2 into pCAGGs by InFusion cloning. GNB1-T2A and GNB2 were amplified from Gi1-CASE, a gift from Gunnar Schulte (Addgene plasmid #168120), and the myc tag sequence was added into primers.
pCAGGS-GNB1-T2A-mApple-GNG2	This study: Insertion of GNB1-T2A, mApple (from pmApple-N1), and GNG2 into pCAGGs by InFusion cloning. GNB1-T2A and GNG2 were amplified from Gi1-CASE, a gift from Gunnar Schulte (Addgene plasmid #168120).
pmApple-N1	From Shaner et al. *Nat. Methods* 2008, 10.1038/nmeth.1209.
mApple-EEA1	This study: replacement of dsRed2 in pDsRed2-EEA1 (from R. Irannejad) with mApple (from pmApple-N1) by InFusion cloning (Takara Bio).
GNB1-P2A-GNG1-IRES-GNAS-LgBit	This study: Deletion of Venus from GNG1 in GNB1-T2A-cpVenus-GNGT1-IRES-GNAS (Blythe and von Zastrow *Nat. Chem. Biol.* 2024, 10.1038/s41589-023-01412-4) by KLD cloning (NEB)) followed by replacement of residues 72-81 with LgBit sequence from pCAGGS_LgBit-CAAX by InFusion cloning (Takara Bio).
pmApple-N1-SmBit(114)-mApple-CAAX	This study: Insertion of N-terminal SmBit(114) tag (Dixon et al. *ACS Chem. Biol.* 11, 10.1021/acschembio.5b00753, sequence added into primers) into pmApple-N1 by KLD cloning, followed by insertion of C-terminal CAAX sequence from pCAGGS-LgBit-CAAX by InFusion cloning (Takara Bio).
pcDNA3.1_SmBiT(114)-miniGs	Blythe and von Zastrow *Nat. Chem. Biol.* 2024, 10.1038/s41589-023-01412-4.
pcDNA3.1_Nb37-SmBiT(101)	Blythe and von Zastrow *Nat. Chem. Biol.* 2024, 10.1038/s41589-023-01412-4.
pcDNA3-SmBit(114)-mApple-KB1691	This study: Insertion of synthesized SmBit(114)-mApple-KB1691 sequence (Twist Biosciences, KB1691 sequence from Janicot et al. *Cell* 2024

	10.1016/j.cell.2024.01.028) into pcDNA3 by InFusion cloning (Takara Biosciences).
pCAGGS_LgBiT-CAAX	From Xu et al. *Nat Chem. Biol.* 2022 10.1038/s41589-021-00930-3.
pCAGGS_endofin-LgBiT	From Xu et al. *Nat Chem. Biol.* 2022 10.1038/s41589-021-00930-3.
pmApple-Arr3-mApple	From Eichel et al. *Nat. Cell Biol.* 2016 10.1038/ncb3307.
pcDNA3.1_SS-HaloTag-VIPR1	Blythe and von Zastrow *Nat. Chem. Biol.* 2024, 10.1038/s41589-023-01412-4.
pcDNA_SS-HaloTag-A_{2B}R	From Blythe et al. b*ioRxiv* 2025 10.1101/2025.02.24.639927.
pcDNA3.1-p63RhoGEF-SmBit(114)	This study: replacement of Nb37 in pcDNA3.1-Nb37(114) (Blythe and von Zastrow Nat. Chem. Biol. 2024, 10.1038/s41589-023-01412-4) with p63RhoGEF residues 295-502 (from Avet et al. *eLife* 2022, 10.7554/eLife.74101), synthesized by Twist Biosensors).
pCMV-Dest_mCherry	Blythe and von Zastrow *Nat. Chem. Biol.* 2024, 10.1038/s41589-023-01412-4.
pCMV-Dest_mCherry-Dyn1K44E	Blythe and von Zastrow *Nat. Chem. Biol.* 2024, 10.1038/s41589-023-01412-4.
pcDNA3-EGFP-GNAS pinned	This study: replacement of amino acids 4 to 6 (LGN) in $G\alpha_s$ with the corresponding sequence in $G\alpha_{i1}$ (TLS).

Supplementary Table 2: CRISPR Knockout Reagents

Target	Cell line(s)	sgRNA sequence 1	sgRNA sequence 2	Forward PCR validation primer	Reverse PCR validation primer	Alternate forward PCR validation primer	Alternate reverse PCR validation primer
GNAS	$G\alpha_s$ KO1 & 2, $G\alpha_{s/olf}$ DKO	GGGCACCAGGUUGCUCAUGG	GGGUUGGCCAGCUCCACGGG	GCATGCAACTTCTGGTACAGTC	GCTCATCCATCTTGAACAAAGCCC	AAGTGTTAGTATGTAGTGTGGG	CATACAGTTTTGTCCTAAGATG
GNAL	$G\alpha_{s/olf}$ DKO	UAUAAUACCUCCAGUUCCGC	UGGUUUUCAGGGUUGGCCAG	CTAGGTCAGTGCTGTAGCTGG	CGCCCCTGGCCGTAGTTAAT	TGATACTGACCTCTAGTGAAAC	TGTATTTTTCGTAGAGATGG

Two primer pairs per gene were used due to inconsistency of amplification efficiency of each reaction.

Supplementary Table 3: CRISPR NGS Primers

Target	Forward primer	Reverse primer
GNAS	ACACTCTTTCCCTACACGACGCTCTTCCGATCT GATGTCTTTATGAAAGCAGTAC	GACTGGAGTTCAGACGTGTGCTCTTCCGATCTCTG TCACTCATGTTCCTATATG

A nested PCR strategy was used with initial amplification with alternate primers in Supplementary Table 2 and a second amplification with primers above.
